# Factors Associated with Treatment Outcomes Among Children and Adolescents Living with HIV Receiving Antiretroviral Therapy in Central Kenya

**DOI:** 10.1089/aid.2021.0112

**Published:** 2022-06-06

**Authors:** Angela McLigeyo, Paul Wekesa, Kevin Owuor, Jonathan Mwangi, Linda Isavwa, Immaculate Mutisya

**Affiliations:** ^1^ Centre for Health Solutions—Kenya (CHS), Nairobi, Kenya.; ^2^ Division of Global HIV and TB, Centers for Disease Control and Prevention, Nairobi, Kenya.

**Keywords:** adolescents, antiretroviral therapy, HIV positive, Kenya, pediatric, treatment outcomes

## Abstract

Expanded access to HIV treatment services has improved outcomes for children and adolescents living with HIV in Kenya. Minimal data are available on these outcomes. We describe temporal trends in outcomes for children and adolescents initiating antiretroviral therapy (ART) from 2004 to 2014 at sites supported by Centre for Health Solutions—Kenya, in central Kenya. We retrospectively analyzed data from children 0–9 years of age (*n* = 3,519) and adolescents 10–19 years of age (*n* = 1,663) living with HIV, who newly initiated ART at 47 health facilities in central Kenya. Year cohorts were analyzed from the Comprehensive Patient Application Database (CPAD) and International Quality Care (IQCare) electronic medical databases, including temporal trends in outcomes and associated factors using multivariable competing risk regression analysis. There were more girls (2,453 [52.7%]) than boys, with most enrolled at World Health Organization (WHO) stage II (1,813 [37.7%]) or III disease (1,694 [35.1%]). Most of the children and adolescents (4,431 [96.4%]) did not have tuberculosis (TB) symptoms. Cumulative lost to follow-up (LTFU) incidence at 6, 12, 24, and 36 months were 5.0%, 9.9%, 22.9%, and 33.1%, respectively. Cumulative mortality incidence at 6, 12, 24, and 36 months were 0.7%, 1.0%, 1.2%, and 1.5%, respectively. The incidence of LTFU was higher among female children and adolescents, those initiated on tenofovir-based regimens, and those with presumptive TB symptoms. Mortality risk was higher among those with WHO stage III or IV disease, and children and adolescents on TB treatment or who had presumptive TB. Enrollment occurred at a young age and pediatric-friendly ART regimens were initiated at earlier WHO stages implying effective early infant diagnosis and treatment for all strategies, resulting in improved treatment outcomes. The higher retention rates in recent years as well as the lower retention after many years of follow-up underscore the importance of implementing longitudinal follow-up strategies targeting this population.

## Introduction

In 2019, ∼1.5 million people in Kenya were living with HIV, including 110,000 children younger than 15 years^1^ with 68.0% of these receiving antiretroviral therapy (ART). An estimated 4,333 children younger than 15 years died of AIDS, and an estimated 2,275 adolescents 10–19 years of age died in 2019.^1^

Mother-to-child HIV transmission (MTCT) is the primary mode of infection for children living with HIV, who typically receive a diagnosis early in life. In southern Africa, median age at enrolment into HIV services for children, including those diagnosed through prevention of mother-to-child transmission programs, has been reported to be less than 3.3 years, with equal distribution between boys and girls.^2^

In 2005, Kenya ART guidelines recommended switching from a combination of stavudine (D4T) and lamivudine (3TC) to a combination of zidovudine (AZT) and lamivudine as the preferred first-line nucleoside reverse transcriptase inhibitor regimen for children and adolescents. However, it took several years to phase out stavudine-based ART regimens. In 2011, guidelines recommended abacavir (ABC)-based regimens as the preferred first-line treatment for children with zidovudine-based regimens as the preferred alternative; tenofovir (TDF) was also recommended as the first-line regimen for adolescents 10–19 years of age, who weigh more than 35 kg.^3,4^ Since 2014, Kenyan treatment guidelines have recommended ART initiation for all children younger than 10 years regardless of World Health Organization (WHO) clinical stage.^3^

Retention of children in HIV services is an important measure of the success of a pediatric HIV program. Few studies have examined temporal trends in the characteristics and outcomes of children initiating ART in Kenya, hence justifying this analysis. As HIV diagnoses and access to ART have increased, it is important to analyze the treatment outcomes of children receiving ART to help improve pediatric HIV programming.

## Materials and Methods

### Study setting

The study was conducted at all 47 government-owned health facilities in the former central province of Kenya, which provide HIV services to children and adolescents and are supported by Center for Health Solutions–Kenya (CHS), through funding from the U.S. President's Emergency Plan for AIDS/Centers for Disease Control and Prevention (PEPFAR/CDC). Per the national HIV treatment guidelines, CHS HIV services included providing HIV testing for children, conducting clinical evaluations, obtaining baseline CD4 cell counts/percentages, and treatment monitoring through viral load testing.

All children are screened for tuberculosis (TB) symptoms: all children received cotrimoxazole to prevent opportunistic infections, and those with no presumptive or active TB also received isoniazid prophylaxis to prevent TB. CHS facilities also provide adherence support, including HIV education, pre-ART counseling and education, adherence counseling at ART initiation and at every visit, and disclosure support for both children and caregivers. Further details of CHS projects in Kenya have been described elsewhere.^5–7^

### Study design, population, and sample

We retrospectively analyzed medical records from a cohort (*n* = 5,182) of children 0–9 years of age and adolescents 10–19 years of age living with HIV, who initiated ART between January 2004 and December 2014 and who were followed for treatment outcomes up to February 2017.

### Data sources

We analyzed data collected between January 2004 and December 2014 using the Kenya Ministry of Health Comprehensive Care Clinic patient card (MOH 257). Clinicians captured the data during routine clinical visits, and then trained data clerks uploaded the data into the Comprehensive Patient Application Database (CPAD) and International Quality Care (IQCare) electronic medical record (EMR) system. These two EMR systems were in use at supported health facilities for patient-data capture and management. Data collected in the two EMR systems were subject to routine review for errors and completeness. The study team prepared the analysis dataset from data extracted from the CPAD and IQCare EMR.

### Variables

Baseline characteristics collected included age at enrollment, sex, WHO clinical stage, date of ART initiation, regimen at ART initiation, and TB status. The backbone regimens at ART initiation containing ABC, AZT, D4T, and TDF were mutually exclusive. Retention was defined as children who were enrolled into ART and who were alive and active in the clinic (not lost to follow-up [LTFU] or dead, as indicated in the patient's chart) as of February 2017. LTFU was determined when the interval between the last clinic visit registered as attended in the database was more than 90 days by time of analysis.^8,9^ Transfer-out status was determined based on documentation in the patient charts. Patients who transferred out were included as active patients in our analysis.

Time to LTFU or mortality outcome was recorded in months. Patients still active in care were censored at 36 months of follow-up. Those who transferred out to other facilities were considered active, but censored at their transfer-out date to utilize their information up to that point. No sign of TB was defined as patients screening negative using the ministry of health symptomatic TB screening tool and presumptive TB was defined as patients screening positive for TB using the symptomatic screening tool, but with pending confirmatory investigations for TB, while presence of TB was defined as patients with confirmed TB based on positive GeneXpert, TB lipoarabinomannan, and/or chest X-ray.

### Ethics

Local ethical approval was obtained from Kenyatta National Hospital—University of Nairobi Ethics Review Committee (P339/06/2013). This study was also reviewed according to the U.S. Centers for Disease Control and Prevention human research protection procedures and was determined to be research, but the CDC investigators did not interact with human subjects or have access to identifiable data or specimens for research purposes. As this research used available retrospective data, consent from study participants was waived. All study procedures were done in accordance with Kenya Government, Ministry of Health, CDC and local IRB regulations.

### Data analysis

Descriptive analysis of demographic and baseline clinical characteristics was done using counts and percentages for all categorical variables. Median and interquartile intervals (IQIs) were presented for time to treatment outcome event. To assess temporal trends in outcomes and characteristics across the ART cohort years, we used the trend function, which is a modified Wilcoxon rank-sum nonparametric test for trends across ordered groups with corresponding *p* values. The person years of observation (pyo) were also calculated and used to determine the LTFU and mortality rates per 100 pyo.

Competing risk regression analysis (Fine and Gray modeling) was done to assess the factors associated with time on ART to LTFU or mortality among the children and adolescents.^10,11^ Mortality outcome was treated as a competing event for being LTFU and likewise, LTFU was treated as a competing event for mortality.

The following variables had missing data: regimen (9.8%), WHO Stage (7.1%), and TB Status (11.3%). CD4 data were missing for over 60% of the subjects and therefore were not included. We assumed the data were missing at random and that missing values could be accounted for by complete measured covariates based on Little's missing completely at random and covariate-dependent missing tests. To deal with missing data, we estimated all the regression models after multiple imputations of missing values using chained equations.^12^ Ten imputed datasets were then used to calculate mean estimates of covariates of interest using Rubin's rules during the regression analysis.^12^ We assessed the observed and imputed values using diagnostic plots to ensure the imputations were reasonable. A sensitivity analysis was done, excluding transfer outs, and this did not change results.

The multivariate models included *a priori* all demographic and clinical characteristics collected. To account for any variability in outcomes by year of ART initiation, we also adjusted for the clustering within the patients' cohort year. The estimated cumulative incidence of mortality and LTFU was based on the multivariable models. The cumulative incidence function plots and subhazard ratios (sHR 95% confidence intervals [CIs]) are presented. Model assumptions were assessed for and met. Statistical significance was evaluated at 5.0% level. All analyses were done using Stata, version 15.1 (StataCorp 2017. Stata Statistical Software, Release 15; College Station, TX).

## Results

### Characteristics of study participants

**FIG. 1.**
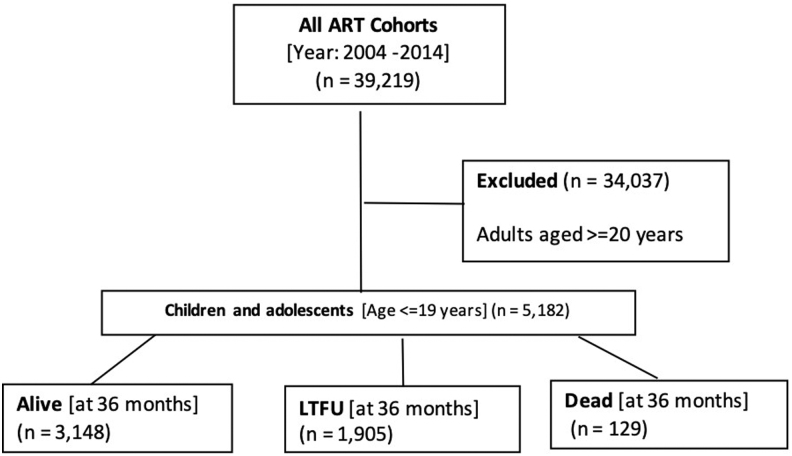
Flow diagram of children and adolescents initiating ART in central Kenya (2004–2014). ART, antiretroviral therapy.

The cohort analysis included 5,182 children and adolescents (Fig. 1). Most of the cohort were children (3,519 [67.9%]) at ART initiation (Table 1). Overall, our cohort included slightly more girls (2,729 [52.7%]) than boys. Most of the patients initiated zidovudine-based (1,827 [39.1%]) or ABC-based regimens, (1,506 [32.2%]). Most children and adolescents had WHO stage II (1,813 [37.7%]) or III (1,694 [35.2%]) disease at ART initiation.

**Table 1.** Characteristics of Children and Adolescents Who Initiated Antiretroviral Therapy in Central Kenya (2004–2014)

ART cohort	2004	2005	2006	2007	2008	2009	2010	2011	2012	2013	2014	Total
*n*	36	138	413	608	662	832	641	586	425	368	473	5*,*182
Age, *n* (%)												
0–4 (*n* = 1,815)	7 (19.4)	43 (31.2)	100 (24.2)	208 (34.2)	230 (34.7)	334 (40.1)	236 (36.8)	213 (36.3)	162 (38.1)	127 (34.5)	155 (32.8)	1,815 (35.0)
5–9 (*n* = 1,704)	22 (61.1)	51 (37.0)	184 (44.6)	212 (34.9)	234 (35.3)	261 (31.4)	213 (33.2)	171 (29.2)	118 (27.8)	96 (26.1)	142 (30.0)	1,704 (32.9)
10–14 (*n* = 997)	4 (11.1)	27 (19.6)	86 (20.8)	130 (21.4)	122 (18.4)	164 (19.7)	112 (17.5)	109 (18.6)	88 (20.7)	71 (19.3)	84 (17.8)	997 (19.2)
15–19 (*n* = 666)	3 (8.3)	17 (12.3)	43 (10.4)	58 (9.5)	76 (11.5)	73 (8.8)	80 (12.5)	93 (15.9)	57 (13.4)	74 (20.1)	92 (19.5)	666 (12.9)
Trend test, *p* value = .099												
Sex, *n* (%)												
Male (*n* = 2,453)	19 (52.8)	54 (39.1)	193 (46.7)	287 (47.2)	328 (49.5)	452 (54.3)	300 (46.8)	272 (46.4)	216 (50.8)	148 (40.2)	184 (38.9)	2,453 (47.3)
Female (*n* = 2,729)	17 (47.2)	84 (60.9)	220 (53.3)	321 (52.8)	334 (50.5)	380 (45.7)	341 (53.2)	314 (53.6)	209 (49.2)	220 (59.8)	289 (61.1)	2,729 (52.7)
Trend test, *p* value = .006^*^												
Regimen, *n* (%)												
D4T (*n* = 1,103)	27 (79.4)	92 (68.1)	235 (58.2)	281 (48.5)	251 (38.9)	164 (24.0)	47 (8.9)	5 (1.0)	1 (0.2)	0 (0.0)	0 (0.0)	1,103 (23.6)
AZT (*n* = 1,827)	7 (20.6)	40 (29.6)	164 (40.6)	285 (49.2)	360 (55.8)	266 (38.9)	195 (36.8)	182 (38.0)	128 (32.0)	113 (31.7)	87 (20.2)	1,827 (39.1)
TDF (*n* = 239)	0 (0.0)	1 (0.7)	3 (0.7)	1 (0.2)	4 (0.6)	4 (0.6)	18 (3.4)	46 (9.6)	37 (9.2)	51 (14.3)	74 (17.2)	239 (5.1)
ABC (*n* = 1,506)	0 (0.0)	2 (1.5)	2 (0.5)	12 (2.1)	30 (4.7)	249 (36.5)	270 (50.9)	246 (51.4)	234 (58.5)	192 (53.9)	269 (62.6)	1,506 (32.2)
Trend test, *p* value <.001^*^												
WHO stage, *n* (%)												
Stage I (*n* = 1,050)	6 (20.0)	7 (5.4)	35 (10.0)	41 (7.8)	79 (13.1)	145 (18.4)	153 (24.7)	145 (25.6)	116 (29.4)	130 (36.6)	193 (42.8)	1,050 (21.8)
Stage II (*n* = 1,813)	0 (0.0)	27 (20.9)	85 (24.2)	195 (36.9)	206 (34.2)	311 (39.6)	268 (43.3)	240 (42.4)	173 (43.8)	132 (37.2)	176 (39.0)	1,813 (37.7)
Stage III (*n* = 1,694)	18 (60.0)	69 (53.5)	177 (50.4)	249 (47.2)	276 (45.8)	302 (38.4)	184 (29.7)	172 (30.4)	86 (21.8)	86 (24.2)	75 (16.6)	1,694 (35.2)
Stage IV (*n* = 256)	6 (20.0)	26 (20.2)	54 (15.4)	43 (8.1)	42 (7.0)	28 (3.6)	14 (2.3)	9 (1.6)	20 (5.1)	7 (2.0)	7 (1.6)	256 (5.3)
Trend test, *p* value <.001^*^												
TB status, *n* (%)												
No TB signs (*n* = 4,431)	27 (90.0)	108 (92.3)	348 (95.3)	511 (94.3)	565 (95.1)	711 (97.0)	553 (96.8)	510 (97.5)	374 (97.4)	311 (98.4)	413 (97.9)	4,431 (96.4)
Presumptive TB (*n* = 42)	0 (0.0)	3 (2.6)	5 (1.4)	6 (1.1)	9 (1.5)	6 (0.8)	7 (1.2)	2 (0.4)	0 (0.0)	1 (0.3)	3 (0.7)	42 (0.9)
On TB TX (*n* = 124)	3 (10.0)	6 (5.1)	12 (3.3)	25 (4.6)	20 (3.4)	16 (2.2)	11 (1.9)	11 (2.1)	10 (2.6)	4 (1.3)	6 (1.4)	124 (2.7)

Trend test, *p* value <.001^*^.

ART, antiretroviral therapy; ABC, abacavir; TB, tuberculosis; TDF, tenofovir.

The proportion of children and adolescents with WHO stage I or II disease at ART initiation increased over the study period, from a low of 6 (20%) in 2004 to a peak of 369 (81.8%) in 2014, while the proportion of those with WHO stage III or IV disease at ART initiation decreased over time, from a high of 80.0% (24) in 2004 to a low of 18.2% (82) in 2014. Most of the children and adolescents (4,431 [96.4%]) did not have signs of TB. This was similar across all the year cohorts: 27 (90.0%) in 2004 and 413 (97.9) in 2014. Overall, 124 (2.7%) of children and adolescents were receiving TB treatment; however, TB treatment rates at ART initiation were higher in earlier cohorts (3 [10.0%] in 2004) compared to later cohorts (6 [1.4%] in 2014; Table 1).

### Treatment outcomes

Overall LTFU was 36.8% (1,905) and mortality was 2.5% (129), respectively. The median time on follow-up was 16.00 months (IQI: 8.00; 25.00) among those LTFU and 4.00 months (IQI: 1.00; 15.00) among those who died (Table 2). There were 1,781 cases of those LTFU and 105 mortality cases observed over 12,153.25 person years at risk. Overall incidence rate (IR) of being LTFU and mortality was 14.65 (95% CI: 13.99–15.35) and 0.86 (95% CI: 0.71–1.05) per 100 pyo.

**Table 2.** Treatment Outcomes of Children and Adolescents Initiating Antiretroviral Therapy in Central Kenya (2004–2014)

36-month follow-up ART outcome	Total	LTFU	Dead	Censored (Retained)
*n* (%)	5*,*182 (100.0)	1*,*905 (36.8)	129 (2.5)	3*,*148 (60.7)
Age, *n* (%)				
0–4 (*n* = 1,815)	1,815 (35.0)	694 (36.4)	41 (31.8)	1,080 (34.3)
5–9 (*n* = 1,704)	1,704 (32.9)	558 (29.3)	43 (33.3)	1,103 (35.0)
10–14 (*n* = 997)	997 (19.2)	341 (17.9)	33 (25.6)	623 (19.8)
15–19 (*n* = 666)	666 (12.9)	312 (16.4)	12 (9.3)	342 (10.9)
Sex, *n* (%)				
Male (*n* = 2,453)	2,453 (47.3)	817 (42.9)	75 (58.1)	1,561 (49.6)
Female (*n* = 2,729)	2,729 (52.7)	1,088 (57.1)	54 (41.9)	1,587 (50.4)
Regimen, *n* (%)				
D4T (*n* = 1,103)	1,103 (23.6)	315 (18.7)	45 (40.2)	743 (25.8)
AZT (*n* = 1,827)	1,827 (39.1)	539 (32.0)	39 (34.8)	1,249 (43.4)
TDF (*n* = 239)	239 (5.1)	161 (9.5)	4 (3.6)	74 (2.6)
ABC (*n* = 1,506)	1,506 (32.2)	672 (39.8)	24 (21.4)	810 (28.2)
WHO Stage, *n* (%)				
Stage I (*n* = 1,050)	1,050 (21.8)	478 (27.0)	9 (7.6)	563 (19.2)
Stage II (*n* = 1,813)	1,813 (37.7)	666 (37.7)	33 (28.0)	1,114 (38.1)
Stage III (*n* = 1,694)	1,694 (35.2)	535 (30.3)	58 (49.2)	1,101 (37.6)
Stage IV (*n* = 256)	256 (5.3)	89 (5.0)	18 (15.3)	149 (5.1)
TB status, *n* (%)				
TB status, *n* (%)	4,431 (96.4)	1,592 (94.3)	85 (74.6)	2,754 (98.5)
No TB signs (*n* = 4,431)	42 (0.9)	17 (1.0)	11 (9.6)	14 (0.5)
Presumptive TB (*n* = 42)	124 (2.7)	79 (4.7)	18 (15.8)	27 (1.0)
ART Cohort, *n* (%)				
2004 (*n* = 36)	36 (0.7)	13 (0.7)	0 (0.0)	23 (0.7)
2005 (*n* = 138)	138 (2.7)	30 (1.6)	2 (1.6)	106 (3.4)
2006 (*n* = 413)	413 (8.0)	82 (4.3)	11 (8.5)	320 (10.2)
2007 (*n* = 608)	608 (11.7)	168 (8.8)	29 (22.5)	411 (13.1)
2008 (*n* = 662)	662 (12.8)	150 (7.9)	24 (18.6)	488 (15.5)
2009 (*n* = 832)	832 (16.1)	188 (9.9)	27 (20.9)	617 (19.6)
2010 (*n* = 641)	641 (12.4)	158 (8.3)	16 (12.4)	467 (14.8)
2011 (*n* = 586)	586 (11.3)	155 (8.1)	8 (6.2)	423 (13.4)
2012 (*n* = 425)	425 (8.2)	132 (6.9)	7 (5.4)	286 (9.1)
2013 (*n* = 368)	368 (7.1)	358 (18.8)	3 (2.3)	7 (0.2)
2014 (*n* = 473)	473 (9.1)	471 (24.7)	2 (1.6)	0 (0.0)
Months to event, median (IQI)	36.00 (20.00; 36.00)	16.00 (8.00; 25.00)	4.00 (1.00; 15.00)	36.00 (36.00; 36.00)

LTFU, lost to follow-up; IQI, interquartile interval.

**FIG. 2.**
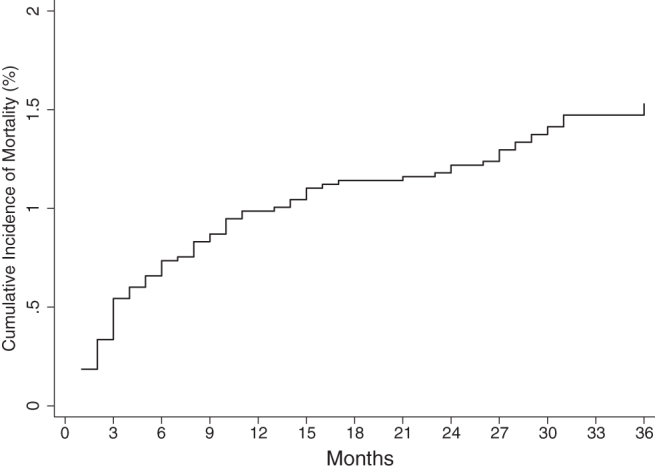
Overall cumulative incidence of mortality among children and adolescents initiating ART in central Kenya (2004–2014).

**FIG. 3.**
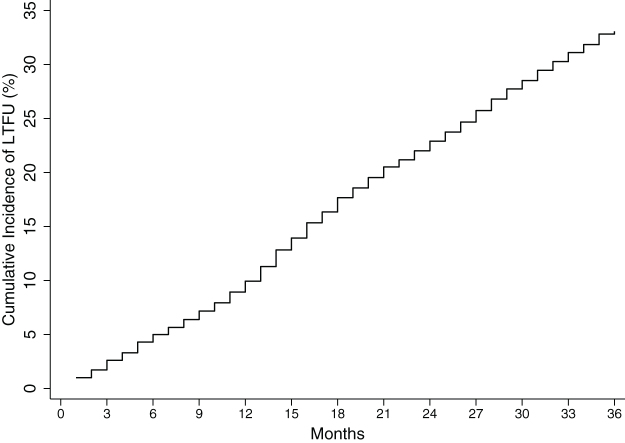
Overall cumulative incidence of being lost to follow-up among children and adolescents initiating ART in central Kenya (2004–2014).

The rate of being LTFU was highest between 13 and 24 months and mortality rate within the first 6 months of follow-up; 17.22 (95% CI: 15.98–18.55) per 100 pyo and 2.09 (95% CI: 1.59–2.76) per 100 pyo, respectively. Overall cumulative incidence of mortality as estimated by the regression model at 6, 12, 24, and 36 months was 0.7%, 1.0%, 1.2%, and 1.5%, respectively (Table 3 and Fig. 2). The overall cumulative incidence of being LTFU as estimated by the regression model was 5.0% at 6 months, 9.9% at 12 months, 22.9% at 24 months, and 33.1% at 36 months of follow-up (Table 3 and Fig. 3).

**Table 3.** Incidence Rate of Being Lost to Follow-Up and Mortality of Children and Adolescents Initiating Antiretroviral Therapy in Central Kenya (2004–2014).

Time (months)	pyo	No. of events (*n*)	Rate per 100 pyo (95% CI)	Cumulative incidence (%)^a^
LTFU outcome				
6 months	2,435.25	305	12.52 (11.19–14.01)	5.0
12 months	2,284.17	275	12.04 (10.70–13.55)	9.9
24 months	4,019.33	692	17.22 (15.98–18.55)	22.9
36 months	3,414.5	509	14.91 (13.67–16.26)	33.1
Total	12,153.25	1,781	14.65 (13.99–15.35)	33.1
Mortality Outcome				
6 months	2,435.25	51	2.09 (1.59–2.76)	0.7
12 months	2,284.17	16	0.70 (0.43–1.14)	1.0
24 months	4,019.33	14	0.35 (0.21–0.59)	1.2
36 months	3,414.5	24	0.70 (0.47–1.05)	1.5
Total	12,153.25	105	0.86 (0.71–1.05)	1.5

^a^ Estimated based on the cumulative incidence function of the multivariable regression model.

CI, confidence interval; pyo, person years of observation.

### Associations between patient characteristics and mortality

**FIG. 4.**
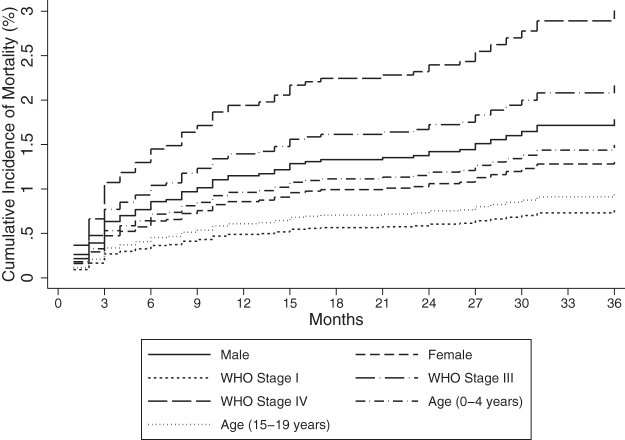
Cumulative incidence of mortality by sex, age, and WHO stage among children and adolescents initiating ART in central Kenya (2004–2014).

The univariable and multivariable models for the risk of mortality are presented in Table 4. On multivariable analysis, there was significantly lower risk of mortality among children and adolescents 10–19 years of age compared to those 0–4 years of age, (sHR, 0.56 [95% CI: 0.38–0.81]). Compared with those classified as WHO stage I disease, the risk of mortality for those with WHO stage III and IV disease was significantly over twofold higher, (sHR, 2.44 [95% CI: 1.15–5.16]) and (sHR, 3.62 [95% CI: 1.20–10.89]), respectively. Compared to those with no sign of TB, children and adolescents on TB treatment and had presumptive TB had significantly higher risk of mortality (aSHR, 11.89 [95% CI: 5.51–25.65]), and (sHR, 5.76 [95% CI: 2.82–11.76]), respectively (Table 4 and Fig. 4).

**Table 4.** Regression Analysis—Mortality Risk Among Children and Adolescents Initiating Antiretroviral Therapy in Central Kenya (2004–2014)

Outcome: mortality	Univariable results		Multivariable results	
Imputed models	sHR (95% CI)	*p*	sHR (95% CI)	*p*
Age groups, years				
0–4	Ref.		Ref.	
5–9	1.08 (0.63–1.87)	.007	0.89 (0.47–1.66)	.703
10–14	1.61 (1.10–2.37)	.015	1.31 (0.83–2.06)	.252
15–19	0.65 (0.41–1.03)	<.001	0.56 (0.38–0.81)	.002
Sex				
Female	Ref.		Ref.	
Male	1.48 (1.02–2.15)	<.001	1.44 (0.93–2.23)	.099
Regimen				
D4T Based	Ref.		Ref.	
AZT Based	0.72 (0.40–1.31)	.916	0.86 (0.45–1.65)	.655
TDF based	0.26 (0.07–0.97)	.004	0.50 (0.13–1.93)	.313
ABC based	0.51 (0.26–0.98)	.221	0.69 (0.31–1.54)	.363
Baseline WHO stage				
Stage I	Ref.		Ref.	
Stage II	2.13 (0.99–4.57)	.902	1.87 (0.91–3.83)	.090
Stage III	3.44 (1.51–7.82)	.599	2.44 (1.15–5.16)	.020
Stage IV	5.49 (2.18–13.86)	.237	3.62 (1.20–10.89)	.022
TB status				
No signs	Ref.		Ref.	
On TB treatment	13.85 (6.60–29.04)	.593	11.89 (5.51–25.65)	<.001
Presumptive TB	6.71 (3.37–13.37)	.001	5.76 (2.82–11.76)	<.001

AZT, zidovudine; D4T, stavudine; sHR, subhazard ratios; TX, treatment; WHO, World Health Organization.

### Associations between patient characteristics and LTFU

**FIG. 5.**
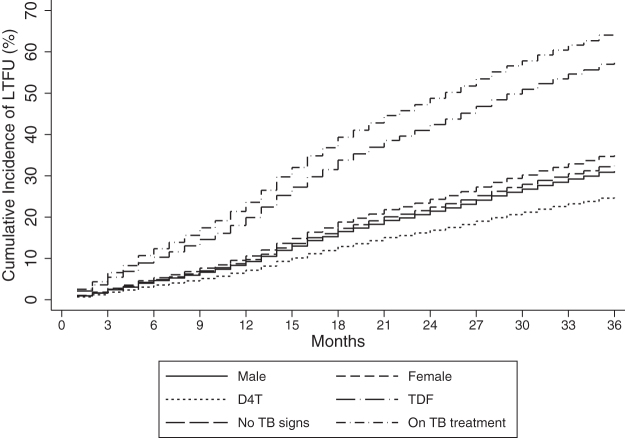
Cumulative incidence of being LTFU by sex, D4T, TDF, and TB status among children and adolescents initiating ART in central Kenya (2004–2014). LTFU, lost to follow-up; TB, tuberculosis; TDF, tenofovir.

The univariable and multivariable models are presented in Table 5. The risk of being LTFU was significantly higher among girls than boys, (aSHR, 1.16 [95% CI: 1.05–1.29]). Children and adolescents who initiated TDF-based ART regimens had significantly higher risk of being LTFU compared to those who initiated stavudine, (aSHR, 2.37 [95% CI: 1.23–4.58]). Children and adolescents with presumptive TB had significantly higher risk of being LTFU compared to those with no sign of TB (aSHR, 2.59 [95% CI: 1.78–3.77]) (Table 5 and Fig. 5).

**Table 5.** Regression Analysis—Lost to Follow-Up Risk Among Children and Adolescents Initiating Antiretroviral Therapy in Central Kenya (2004–2014)

Outcome: LTFU	Univariable results		Multivariable results	
Imputed models	sHR (95% CI)	*p*	sHR (95% CI)	*p*
Age groups, years				
0–4	Ref.		Ref.	
5–9	0.84 (0.75–0.95)	.007	0.93 (0.74–1.17)	.551
10–14	0.91 (0.84–0.98)	.015	1.05 (0.80–1.38)	.735
15–19	1.35 (1.19–1.54)	<.001	1.26 (1.00–1.59)	.051
Sex				
Male	Ref.		Ref.	
Female	1.25 (1.12–1.40)	<.001	1.16 (1.05–1.29)	.003
Regimen				
D4T based	Ref.		Ref.	
AZT based	1.03 (0.64–1.64)	.916	1.02 (0.65–1.59)	.933
TDF based	3.03 (1.43–6.42)	.004	2.37 (1.23–4.58)	.010
ABC based	1.66 (0.74–3.75)	.221	1.64 (0.74–3.66)	.225
Baseline WHO stage				
Stage I	0.98 (0.65–1.46)	.902	0.92 (0.63–1.35)	.676
Stage II	1.16 (0.67–2.02)	.599	1.08 (0.71–1.63)	.722
Stage III	1.56 (0.75–3.25)	.237	1.35 (0.83–2.22)	.228
Stage IV	Ref.		Ref.	
TB status				
No signs	Ref.		Ref.	
On TB treatment	1.22 (0.59–2.53)	.593	1.37 (0.69–2.75)	.372
Presumptive TB	2.30 (1.39–3.79)	.001	2.59 (1.78–3.77)	<.001

AZT, zidovudine; D4T, stavudine; WHO, World Health Organization; TX, treatment.

## Discussion

Most patients were younger than 10 years at enrolment across all the cohorts studied. The higher number of young children enrolled was likely due to easier access to HIV services, young age at HIV diagnosis, and the natural progression of HIV among children (most perinatally infected children present with signs and symptoms of HIV within the first decade of life). Our findings support those of a multicenter study conducted in sub-Saharan Africa, which reported a median age of 4.9 years for children initiating ART.^13^ Studies in sub-Saharan Africa also reported that the age at ART enrolment for children progressively decreased for later cohorts.^14^ Among older children and adolescents, youth-friendly models of care could help increase ART initiation.^15^

Most children and adolescents in our cohort initiated stavudine-based or ABC-based regimens. This finding is expected because of changes in national treatment guidelines recommending simplified and more tolerable antiretroviral agents for children and adolescents.^3,4^

The number of children and adolescents with baseline WHO stage I disease increased in later cohorts, whereas the number of children and adolescents with WHO stage III disease decreased. Our finding supports the results of several studies. A study conducted in four provinces in South Africa reported that the proportion of children with baseline WHO stage III or IV disease decreased from 72.9% to 49.0% between 2006 and 2009.^16^ In Tanzania, a study of 44 HIV clinics between 2005 and 2011 reported a decreasing trend of patients with baseline WHO stage IV disease at ART enrollment.^17^

In contrast, another study reported that 57.0% of children presented at WHO clinical stage III and IV at ART enrolment.^18^ Enrolment into HIV treatment programs at early stages of HIV disease could be due to the expansion of early infant diagnosis and enhanced provider-initiated testing in Kenya.^19,20^ This may also be due to progressively more inclusive ART initiation guidelines.^3,4^

In our study, few children and adolescents had a documented positive screen for TB at the time of ART enrollment, and the number of children and adolescents receiving treatment for TB at ART enrollment decreased in later cohorts. More than half the study population in earlier cohorts had WHO stage III or IV disease; we were not able to determine how many children and adolescents had documented completion of TB treatment before ART enrollment, which could have informed their WHO classification. We also were unable to determine how many children and adolescents received a TB diagnosis while in care. This may partly explain the low number of children and adolescents with documented TB/HIV co-infection in our study.

Another possibility is that the TB symptom screening tool used for HIV-positive patients is highly dependent on provider skill and therefore may have resulted in missed TB diagnoses in this population.^21^ Furthermore, health care workers run busy clinics and may not screen children adequately for TB. Since TB symptoms are nonspecific, symptoms of TB may have been attributed to other diseases, including HIV infection.^22^

To help avoid missing TB diagnoses, clinicians could ensure that patients are screened more thoroughly for TB, due to overlap in the clinical manifestations of both diseases.^23^ There is also need to improve the capacity of ART programs in low-income and middle-income countries to exclude and diagnose TB in children living with HIV. Our finding of high mortality rates among study participants with no sign of TB conflicts with results from other studies in sub-Saharan Africa, which have consistently shown that TB is a leading cause of death among children and adolescents living with HIV.^24^

The conflicting result could be due to missed TB diagnosis using the symptomatic TB screening tool, resulting in more children dying from undiagnosed TB, thus resulting in an underestimation of childhood TB mortality in our study. This is compounded by the fact that current diagnostic tests for TB in children, even when done correctly, underperform and may result in children with TB being misclassified as having no TB.

There were patients in our study classified as having presumptive TB. The presence of presumptive TB cases could be due to delays in conducting confirmatory tests for TB or even challenges in accessing health care. The higher risk of mortality noted among presumptive TB cases could be due to delays in initiating TB treatment in these suspected, but not confirmed TB cases. At the time of the study, Kenyan guidelines did not recommend treatment of presumptive TB cases with anti-TB medicines.

In this study, the overall incidence of LTFU was 36.8% and overall mortality was 2.5% after a median follow-up time of 16 months for being LTFU, which was similar to a study conducted in South Africa that reported a cumulative LTFU incidence of 36% after a median follow-up time of 3.3 years.^25^ Overall IR of being LTFU and mortality was 14.65 and 0.86 per 100 person years compared to IR of LTFU of 10.8 per 100 person years in South Africa.^25^ Overall cumulative incidence of being LTFU and mortality increased with the number of years of follow-up.

Studies conducted among adults also have reported higher incidence of being LTFU in successive years of the ART program.^26–29^

Mortality rate was low in our study. Similarly, other studies have recorded decreased mortality rates in children who initiated ART.^30^ A systematic review in 15 sub-Saharan African countries among 51,619 pediatric patients receiving first-line ART from 2014 to 2018 reported low mortality rates of 3.0%, 5.0%, 6.0%, and 7.0% at 3, 6, 12, and 24 months after ART initiation, respectively.^31^ In contrast, a study in Mozambique reported a high mortality rate (29.0%) among 735 children after 2 years of ART.^31^ These differences could be due to disparities in the data, such as undocumented deaths reported as LTFU.^32^

We found higher risk of death among children 0–4 years of age, those with WHO stage III or IV disease, those on TB treatment, and those with presumptive TB.

Studies elsewhere have identified young age as a risk factor for mortality.^33,34^ In contrast, a study conducted in four HIV programs in Kenya, Uganda, and Malawi reported high mortality rates among children 5–14 years of age compared to those 2–4 years of age.^35^

Similar to our study, WHO stage III and IV disease and TB co-infection are associated with increased likelihood of death.^31,35–37^ The higher risk of mortality in children and adolescents with presumptive TB could be due to delayed TB diagnosis.

In our study, girls, children, and adolescents receiving TDF, and those with presumptive TB had higher risk of being LTFU. Transition of children to adolescents could have resulted in higher risk of being LTFU among those on TDF. Studies have shown greater LTFU during this transition.^38^

Our finding that children with advanced HIV had higher risk of being LTFU could be because of associated opportunistic infections which may result in undocumented deaths.^36,37^ Identifying children who miss scheduled appointments early and developing strategies directed at retaining them in care are critical to improving long-term pediatric HIV outcomes.

Our study had several limitations. The retrospective design of the study could have resulted in missing data that were not possible to retrieve. Poor documentation in patient records resulted in missing data from the electronic database. Some instances of being LTFU could have been patients who enrolled in other facilities following self-referral or could have been unreported deaths, resulting in falsely higher LTFU rates.^38^ Viral load data were not analyzed because routine viral load monitoring was not available in Kenya before 2014. TB data were suboptimal since it did not analyze for diagnosis of TB during follow-up period nor for completion of TB treatment. Furthermore, poor quality screening for TB as well as lack of treatment for presumptive TB could have skewed our outcome data.

## Conclusion

In conclusion, children were enrolled into care at a young age. This implies that early infant diagnostic approaches are effective. Over time, children and adolescents initiated ART at earlier WHO clinical stages, implying that PITC is a useful strategy in implementing prompt HIV testing and treatment of HIV in children and adolescents and should continue to be scaled up. This is especially important because children and adolescents who were enrolled at advanced WHO stages had higher risk of getting LTFU and mortality. The study found that children were switched from initial regimens at enrolment to pediatric friendly regimens in line with both WHO and Kenya HIV guideline recommendations. We did not analyze whether the use of pediatric-friendly regimens resulted in an improvement in early ART initiation.

Retention rates reduced with longer follow-up periods, underscoring the importance of strong systems to ensure longitudinal follow-up of patients on cART. The higher risk of mortality and getting LTFU among children and adolescents with presumptive TB could have been due to delayed TB diagnosis and initiation of TB treatment. Higher risk of mortality among WHO stage III and IV patients also implies the need to establish systems to identify and manage opportunistic infections early in this population. These results are generalizable to other HIV programs within Kenya and sub-Saharan Africa, which implement similar CDC/PEPFAR-funded programs.

## Ethics Approval and Consent to Participate

The Kenyatta National Hospital—University of Nairobi Ethics Review Committee. This study was reviewed according to the U.S. Centers for Disease Control and Prevention human research protection procedures and was determined to be research, but the CDC investigators did not interact with human subjects or have access to identifiable data or specimens for research purposes. As this research was retrospective, consent from study participants was not required.

## PEPFAR/CDC Disclaimer

The findings and conclusions in this publication are those of the authors and do not necessarily represent the official position of the funding agency.
